# A Novel FAM83H Truncation Mutation Disrupts Enamel Mineralization in Autosomal Dominant Hypocalcified Amelogenesis Imperfecta

**DOI:** 10.1002/cre2.70431

**Published:** 2026-08-13

**Authors:** Yue Wang, Hongfei Chen, Jiyong Lai, Xueqing Huang, Yanyu Huang

**Affiliations:** ^1^ Guanghua School and Hospital of Stomatology, Guangdong Provincial Key Laboratory of Stomatology, Institute of Stomatological Research Sun Yat‐sen University Guangzhou Guangdong China; ^2^ Ganzhou Weile Dental Hospital Ganzhou Jiangxi China

**Keywords:** ADHCAI, amelogenesis imperfecta, enamel calcification, FAM83H

## Abstract

**Objectives:**

Autosomal dominant hypocalcified amelogenesis imperfecta (ADHCAI; OMIM#130900) is a hereditary enamel defect caused by truncation mutations in *FAM83H*, though the underlying pathogenic mechanisms remain incompletely understood. This study aimed to identify novel *FAM83H* mutations in a Chinese family with ADHCAI and to investigate their functional consequences on enamel formation.

**Materials and Methods:**

A three‐generation ADHCAI family underwent clinical and genetic evaluation. Whole‐exome sequencing and Sanger sequencing were used for mutation identification. The proband's enamel was analyzed by scanning electron microscopy with energy‐dispersive X‐ray spectroscopy (SEM‐EDX). Structural impacts of the mutation were predicted bioinformatically (PSIPRED, SWISS‐MODEL). Patient‐derived periodontal ligament cells (PDLCs) were assessed by Western blot, immunofluorescence, and quantitative real‐time PCR for key enamel matrix proteins (AMELX, AMBN, ENAM) and osteogenic markers (RUNX2, ALP).

**Results:**

A novel heterozygous nonsense mutation (c.1819G>T, p.(Glu607*)) in *FAM83H* was identified, resulting in a C‐terminally truncated protein lacking 573 residues that mislocalizes from the cytoplasm to the nucleus. SEM‐EDX revealed disorganized enamel ultrastructure, a reduced calcium‐to‐phosphorus ratio, and increased porosity. Functional analysis in PDLCs showed coordinated downregulation of enamel matrix proteins and osteogenic markers, indicating a dual regulatory role for FAM83H in biomineralization.

**Conclusions:**

Our findings are consistent with the “presecretory pathogenesis” model for ADHCAI, wherein nuclear‐mislocalized truncated FAM83H may disrupt enamel mineralization partly through transcriptional suppression of matrix components. This expands the *FAM83H* mutational spectrum, offers preliminary mechanistic insight, and suggests potential targets for pre‐eruptive therapeutic strategies.

## 1 Introduction

Autosomal dominant hypocalcified amelogenesis imperfecta (ADHCAI; OMIM#130900) is a severe inherited enamel defect characterized by soft and fragile enamel, which undergoes rapid post‐eruptive wear and is notably associated with dentin hypersensitivity and significant esthetic impairment (Smith et al. 2017). Although more than 30 truncation mutations in the *FAM83H* gene—the only known causative gene for ADHCAI (Kim et al. 2008)—have been reported, the precise molecular mechanisms linking these mutations to the clinical phenotype remain incompletely elucidated.

FAM83H is a cytoplasmic scaffold protein critical for enamel mineralization. It regulates cytoskeletal organization and casein kinase 1ε (*CSNK1E*) signaling through distinct functional domains (Kuga et al. 2013). Similar scaffolding functions have been proposed for casein kinase 1α (*CSNK1A1*), another potential interaction partner of FAM83H (Kuga et al. 2016). All reported pathogenic mutations cluster within exon 5, which encodes its C‐terminal domain, resulting in truncated proteins. A central paradox in the field arises from contradictory findings in animal models: *Fam83h* knockout mice exhibit normal enamel (Wang et al. 2016), whereas models expressing C‐terminally truncated *Fam83h* recapitulate the human disease phenotype (Zheng et al. 2023). These observations suggest that ADHCAI results not from haploinsufficiency but from dominant‐negative or gain‐of‐function mechanisms driven by aberrant mutant protein behavior.

A critical consequence of truncation is the mislocalization of FAM83H. Recent studies show that truncated FAM83H evades nonsense‐mediated decay and accumulates in the nucleus (Lee et al. 2011), where it impairs the function of wild‐type protein and key partners such as *CSNK1A1* (Kuga et al. 2016). However, the functional consequences of this nuclear translocation on ameloblast biology remain poorly understood. Prevailing hypotheses have emphasized FAM83H's role in post‐secretory structural stabilization, yet emerging *in vitro* evidence suggests that mutant FAM83H can downregulate enamel matrix protein expression (Xie et al. 2023), hinting at a previously unrecognized role during the presecretory phase.

This leads to a fundamental question: how can pre‐eruptive suppression of matrix components be reconciled with the clinically normal enamel thickness? Resolving this discrepancy is essential for advancing our understanding of ADHCAI pathogenesis. We hypothesized that novel *FAM83H* truncation mutations disrupt enamel formation through nuclear mislocalization‐induced transcriptional dysregulation, ultimately producing a qualitatively deficient matrix that is mechanically unsound.

To test this hypothesis, we identified a novel heterozygous nonsense mutation (c.1819G>T, p.(Glu607*)) in a three‐generation Chinese family with ADHCAI. We characterized its effect on protein localization and investigated downstream molecular and ultrastructural consequences. Our results demonstrate that the mutant protein mislocalizes to the nucleus and induces dual suppression of enamel matrix and osteogenic gene programs. These findings suggest a potentially important role for presecretory transcriptional dysregulation in ADHCAI pathogenesis, complementing existing structural models. This perspective offers a potential explanation for certain clinical and pathological features of the disease.

## 2 Materials and Methods

### 2.1 Recruitment of a Family with ADHCAI

A Chinese family diagnosed with ADHCAI was recruited for this study. All participating family members underwent comprehensive oral examinations, including intraoral photography, radiographic assessment, and clinical evaluation. The pedigree was constructed based on detailed medical history and the findings from these oral examinations. Additionally, three healthy volunteers with intact dental enamel, matched for age and sex, were enrolled as the control group. Written informed consent was obtained from all participants. The study was approved by the Ethical Committee of the School and Hospital of Stomatology, Sun Yat‐sen University (Approval No. KQYC‐2023‐39‐02).

### 2.2 Mutation Analysis

Genomic DNA was extracted from 5 mL of peripheral blood collected from each participant using the TIANGEN Blood DNA Mini Kit (TIANGEN, DP304) according to the manufacturer's instructions. Whole‐exome sequencing was performed on genomic DNA from the proband, her affected mother, her affected grandmother, and her unaffected uncle. Exome sequences were captured using the xGen^TM^ Exome Research Panel v2 (Integrated DNA Technologies), and sequencing was subsequently performed on the DNBSEQ‐T7 platform (MGI, RRID: SCR_019287). Data were processed using the analytical software bcbio‐nextgen‐v.1.2.8 (RRID: SCR_015940) and the Genome Analysis Toolkit (GATK v4.1.2, RRID: SCR_001876). Adaptors were removed, and raw reads were aligned to the human reference genome (NCBI GRCh37, reference genome hg19) using the Burrows–Wheeler Aligner (BWA v0.7.17, RRID: SCR_010910). Polymerase chain reaction (PCR) duplicates were removed with Picard (v2.23.8, RRID: SCR_006525, http://broadinstitute.github.io/picard/), and the variant calling was performed using GATK best practices, followed by annotation with ANNOVAR v2018Apr16 (RRID: SCR_012821).

Candidate variants were filtered according to the following criteria: (a) absent or minor allele frequency <0.01 in the 1000 Genomes Variant Database (Phase 3, RRID: SCR_006828), Genome Aggregation Database (gnomAD v2.1.1, RRID: SCR_014964), or NHLBI‐GO Exome Sequencing Project (ESP v6500, RRID: SCR_012761) databases; (b) presence in both the proband and her affected mother; (c) predicted to be deleterious; and (d) phenotypic relevance.

Regions identified as disease‐causing via whole‐exome sequencing were validated by PCR and Sanger sequencing. Primers targeting *FAM83H* (NM_198488.5) were designed using Primer‐BLAST (http://www.ncbi.nlm.nih.gov/tools/primerLblast/). The primers used are listed in Table S1. PCR amplicons were sequenced on an ABI 3500 Genetic Analyzer (PE Applied Biosystems, RRID: SCR_020226) and compared against normal reference *FAM83H* sequences using BLASTN (v2.12.0, RRID: SCR_004870).

### 2.3 Bioinformatic Analysis

Mutation Taster 2021 (RRID: SCR_014216, http://www.mutationtaster.org) was used to evaluate the pathogenic potential of the variant. Secondary and tertiary structures of wild‐type and mutant FAM83H proteins were predicted using the online program PSIPRED Protein Sequence Analysis Workbench (v4.0, RRID:SCR_010879, http://bioinf.cs.ucl.ac.uk/psipred/) and SWISS‐MODEL (v2023, RRID:SCR_018123, https://swissmodel.expasy.org/), respectively.

### 2.4 Histological Characterization

For histological analysis, four third molars extracted from the proband and age‐matched healthy controls were dehydrated through a graded ethanol series (70%–100%), embedded in methyl methacrylate, and sectioned mesiodistally using an EXAKT system (EXAKT 300 CL, EXAKT). Section surfaces were polished, etched with 37% phosphoric acid for 10 s, rinsed thoroughly with copious amounts of water, and coated with palladium before examination under a scanning electron microscopy (SEM, ZEISS GeminiSEM 300, Zeiss, RRID: SCR_018645) at 15 kV. Elemental composition (C, O, P, Ca) was assessed at three sites per sample using energy‐dispersive X‐ray spectroscopy (EDX, Oxford Instruments X‐MaxN).

### 2.5 Cell Isolation and Culture

Periodontal ligament cells (PDLCs) were isolated from the proband's third molar. Similar passages of cells from healthy individuals served as controls. Freshly extracted teeth were rinsed with phosphate‐buffered saline (PBS) for 5 min, and periodontal tissues from the middle third of the root were gently scraped and planted in 25 cm^2^ cell culture dishes and maintained in Dulbecco's modified Eagle's medium (DMEM, Life Technologies Corporation, 11965062, RRID:SCR_022587) supplemented with 10% fetal bovine serum (FBS, Life Technologies Corporation, 10437028, RRID:SCR_022587) and 1% penicillin and streptomycin (Life Technologies Corporation, 15140122, RRID:SCR_022587). The cells were cultured at 37°C in humidified air containing 5% CO_2_, with culture medium changes every 2 days. After reaching confluence, the cells were passaged at a 1:3 ratio. PDLCs at passage 5 were used in subsequent experiments.

### 2.6 Subcellular Localization Assay

Twenty‐four hours after seeding onto sterile, collagen‐coated glass coverslips placed within culture dishes, the cells were washed with PBS three times for 1 min each and then fixed with 4% paraformaldehyde for 20 min. After three 5‐min washes with PBST (PBS + 0.1% Triton X‐100), the cells were blocked with 10% goat serum (Invitrogen, Cat# 16210072, RRID: SCR_022587) for 1 h at room temperature, and incubated with rabbit anti‐FAM83H antibody (Biorbyt, Cat# orb183479; 1:100 dilution) at 4°C overnight followed by Alexa Fluor 488‐conjugated goat anti‐rabbit IgG secondary antibody (Abcam, Cat# ab150077, RRID: AB_2864372; 1:400 dilution) for 1 h at room temperature. The coverslips were then mounted with Fluoroshield^TM^ mounting medium containing 4',6‐diamidino‐2‐phenylindole (DAPI, Abcam, Cat# ab104139, RRID: AB_10889838). The cells in the two groups were then visualized and captured using an Olympus FV3000 confocal microscope (Olympus, RRID: SCR_019236).

### 2.7 RNA Isolation and Quantitative Real‐Time PCR Analysis

After 48 h of culture, total RNA was extracted from the PDLCs using TRIzol reagent (Invitrogen, Cat# 15596026, RRID: SCR_022587) and reverse‐transcribed to cDNA using the PrimeScript^TM^ RT Kit (TaKaRa, Cat# RR037A). Quantitative real‐time PCR (RT–qPCR) was performed to quantify the expression levels of *FAM83H*, amelogenesis‐related genes, and osteogenesis‐related genes using LightCycler 480 (Roche, RRID: SCR_020246). cDNA was used as a template for RT‐qPCR, which was performed according to the manufacturer's protocol with TB Green Premix Ex Taq^TM^Ⅱ (TaKaRa, Cat# RR820A). Primers are listed in Table S2. All experiments were performed in triplicate.

### 2.8 Western Blot Analysis

Cells from the mutant and control groups were harvested after 48 h of culture and homogenized in RIPA lysis buffer (Beyotime Biotechnology, Cat# P0013B) supplemented with protease inhibitors (Thermo Fisher Scientific, Cat# 78430, RRID: SCR_022587). The protein concentrations of the extracted samples were quantified by bicinchoninic acid (BCA) assay (Thermo Fisher Scientific, Cat# 23225, RRID: SCR_022587). Equal amounts of protein (20 µg) from the two groups were resolved by SDS–PAGE, transferred onto polyvinylidene difluoride membranes, and incubated overnight at 4°C with primary antibodies. Then, the blots were incubated with goat anti‐rabbit secondary antibodies conjugated with horseradish peroxidase (HRP, Abcam, Cat# ab205718, RRID: AB_2819160; 1:1000 dilution). The signals were visualized using a chemiluminescence detection system (Thermo Fisher Scientific, Cat# 34580, RRID: SCR_022587) with CL‐XPosure film (Pierce Biotechnology, Cat# 34090). The primary antibodies used were rabbit polyclonal anti‐AMBN antibody (Proteintech, Cat# 26501‐1‐AP, RRID: AB_2884950; 1:1000 dilution), rabbit polyclonal anti‐AMELX antibody (Affinity Biosciences, Cat# DF13729; 1:1000 dilution), rabbit polyclonal anti‐ALP antibody (Abcam, Cat# ab229126; 1:1000 dilution), and rabbit polyclonal anti‐FAM83H antibody (Biorbyt, Cat# orb183479; 1:1000 dilution).

### 2.9 Statistical Analysis

Data are represented as means ± standard deviations from three technical replicates per sample. For patient‐derived PDLCs, a single cell line established from the proband was compared with three independently derived PDLC lines from age‐ and sex‐matched healthy controls. All experiments were performed in triplicate. Statistical significance between two groups was determined using two‐tailed Student's *t*‐test. A *p‐*value < 0.05 was considered statistically significant. *p < 0.05, **p < 0.01, ***p < 0.001, and ****p < 0.0001.

## 3 Results

### 3.1 Clinical Phenotypic Characterization

**FIGURE 1:**
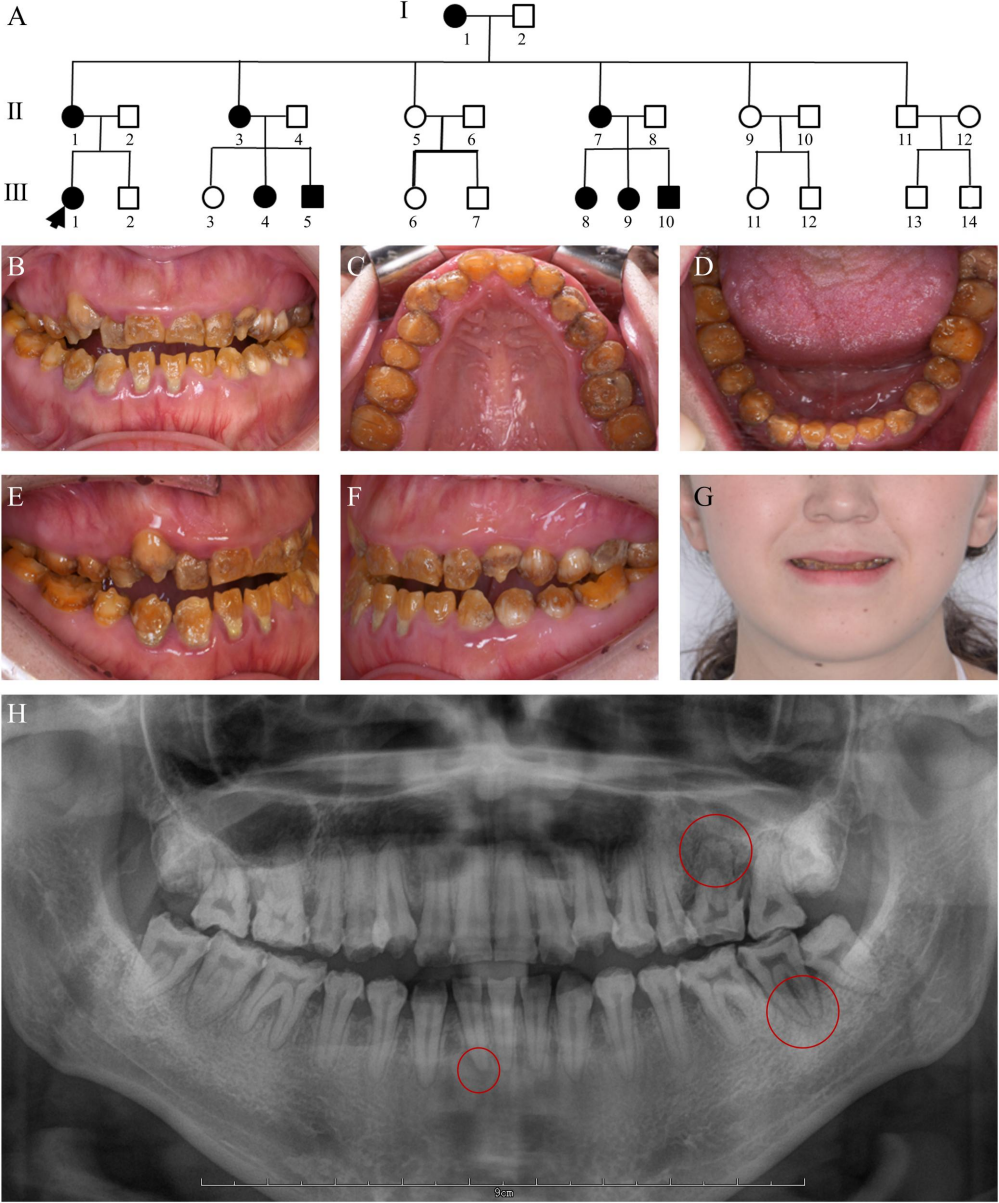
Clinical and radiographic phenotypes of the proband with ADHCAI. (A) Pedigree of the family. Squares represent males and circles represent females. Filled symbols designate affected individuals, while open symbols indicate those not affected. The proband is indicated by a black arrow. (B–F) Intraoral images of the proband (III:1), showing a crossbite, along with severe enamel discoloration and abrasion. (G) Cropped perioral image showing reduced lower facial height of the proband. (H) Panoramic radiograph of the proband, demonstrating reduced enamel density and periapical periodontitis of some teeth (marked by red circles).

In this study, a three‐generation Chinese family presenting with ADHCAI was recruited. The proband (individual III:1 in Figure 1A) was a 20‐year‐old female who sought dental consultation due to severe esthetic impairment. Comprehensive clinical evaluations were performed on the proband and her family, in which 10 of 28 members met the diagnostic criteria for ADHCAI based on their typical phenotype (Figure 1A). The medical history of the family members revealed no other severe systematic diseases. Therefore, ADHCAI was diagnosed on the basis of clinical manifestations and the pattern of inheritance.

The proband exhibited typical severe hypocalcified amelogenesis imperfecta, featuring porous and extremely soft enamel, severely occlusal wear with >50% crown height reduction, rough and irregular tooth surfaces, and exposed dark dentin with brown and black discoloration (Figure 1B–F). Reduced crown dimensions led to loss of proximal and interocclusal spaces. Small regions of normal enamel thickness were still observed along the gingival margins and cusp tips of some molars (Figure 1E,F). The patient presented anterior crowding, functional crossbite, generalized plaque accumulation, and gingival inflammation (Figure 1B,G). Compared with normal teeth, panoramic imaging of the proband demonstrated enamel loss and reduced radiodensity contrast between enamel and dentin; periapical radiolucencies were observed around the apices of teeth #16, #37, and #41. Notably, pulp chamber dimensions remained preserved despite significant attrition (Figure 1H).

### 3.2 Ultrastructure of the Enamel

**FIGURE 2:**
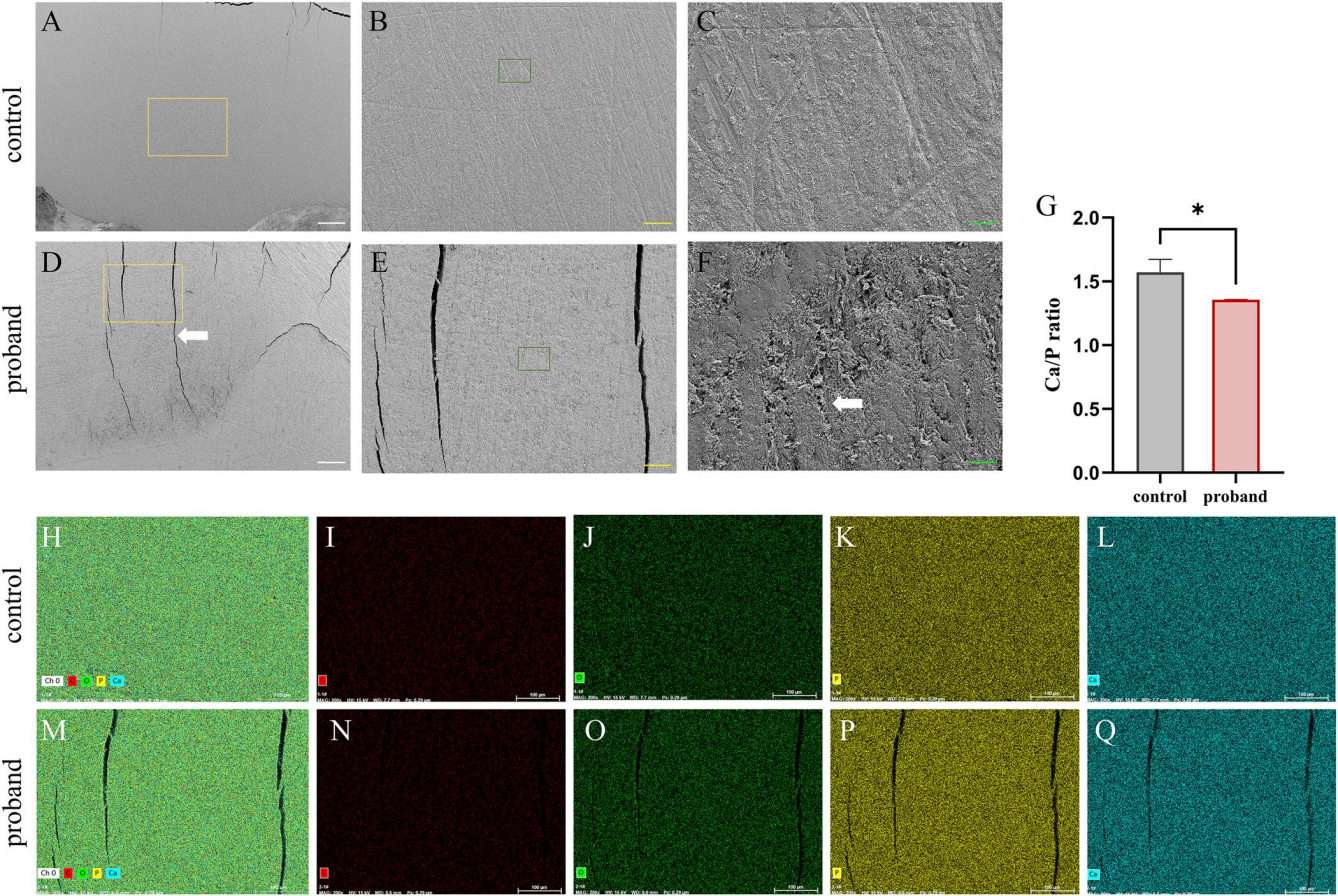
Ultrastructure and elemental mapping of enamel in the proband. (A–C) Scanning electron microscopy (SEM) images of a normal control. Scale bars: 200 μm (white), 50 μm (yellow), and 5 μm (green). (D–F) SEM images of the proband. Multiple enamel lamellae are visible throughout the entire enamel layer (white arrow in D), and the spaces between the enamel rods are widened compared to the normal control (white arrow in F). Scale bars: 200 μm (white), 50 μm (yellow), and 5 μm (green). (G) SEM‐EDS analysis of the Ca/P ratio in normal control (left) and proband (right) enamel. Quantitative measurements revealed a significantly lower ratio in the proband (1.3 ± 0.1) compared to controls (1.5 ± 0.1; two‐tailed Student's *t*‐test: *t*_(4)_ = 2.78 *p* = 0.049). (H–L) Elemental mapping with SEM‐EDX of the normal control. Uniform distributions of C, O, P and Ca are observed. (M–Q) Elemental mapping with SEM‐EDX of the proband, also showing uniform distributions of C, O, P and Ca.

SEM analysis revealed profound structural abnormalities in the proband's enamel. Significant differences were observed between the control (Figure 2A–C) and the proband (Figure 2D–F). Control specimens displayed well‐defined prism structures and intact dentin–enamel connections. In contrast, the proband's enamel exhibited an amorphous microstructure with elevated surface porosity, discontinuous dentin–enamel junctions, and disorganized rod orientation.

EDX demonstrated that the enamel of the proband had a significantly reduced calcium‐to‐phosphorus ratio (Ca/P: 1.3 ± 0.1 vs. 1.5 ± 0.1 in controls; two‐tailed Student's *t*‐test: *t* (4) = 2.78, *p* = 0.049, Figure 2G). Element mapping revealed uniform distributions of C, O, P, and Ca in both the normal control (Figure 2H–L) and the proband (Figure 2M–Q).

### 3.3 Mutation Identification

**FIGURE 3:**
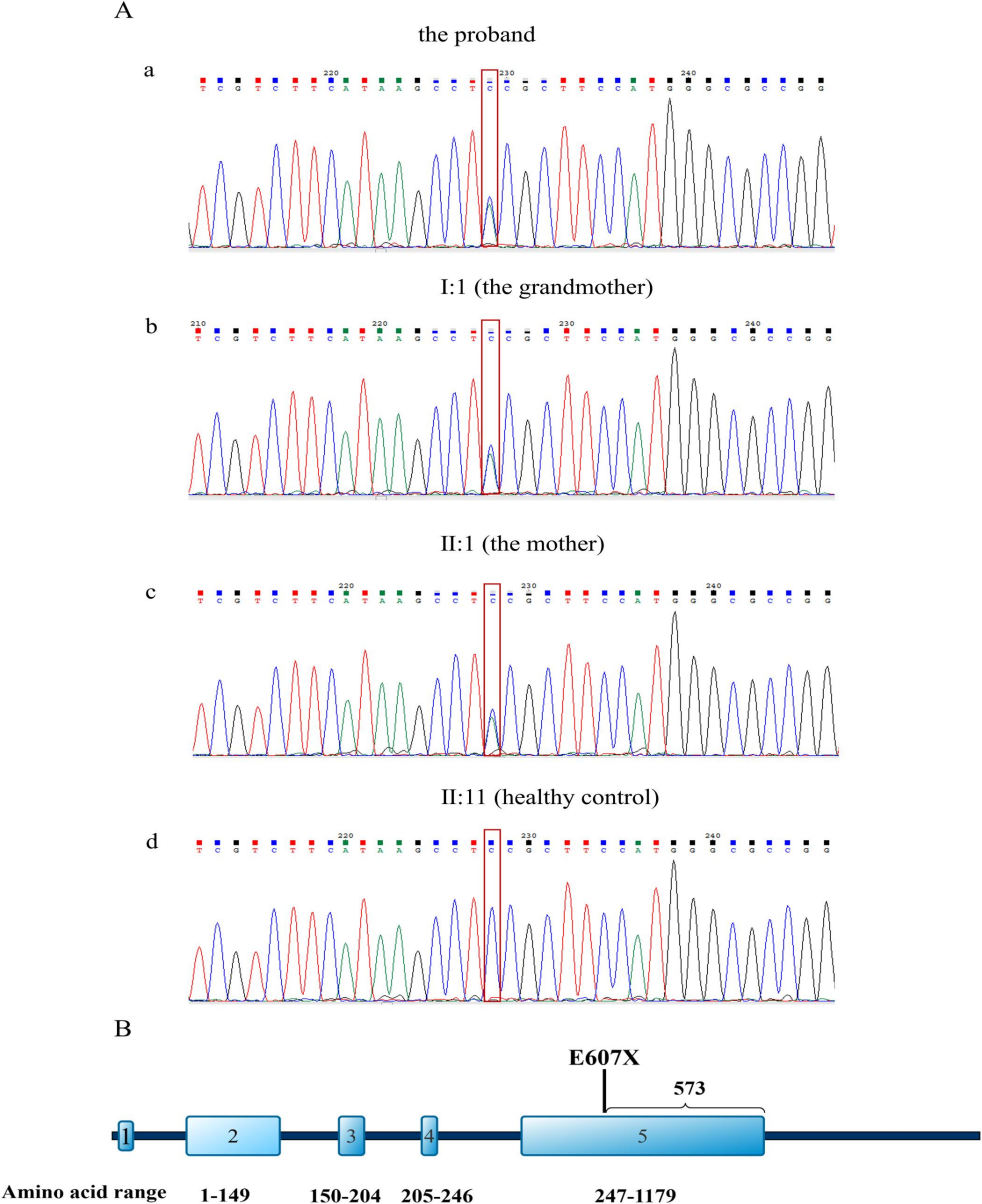
Mutation analysis of the *FAM83H* gene in ADHCAI patients. (A) Sanger sequencing chromatograms showing the heterozygous c.1819G>T mutation. From top to bottom: (a) the proband (III:1, affected), (b) the grandmother (I:1, affected), (c) the mother (II:1, affected), and (d) healthy control (II:11, unaffected family member). Mutated and wild‐type nucleotide residues are highlighted in red boxes. (B) Schematic diagram of the *FAM83H* gene structure containing the annotated mutation identified in this study. The gene diagram shows 5 exons (boxes) and 4 introns. The exon coding regions are shaded. The mutation site is marked by a black line.

To identify mutation sites in the *FAM83H* gene in ADHCAI patients, we analyzed the coding region of *FAM83H*. The original whole‐exome sequencing data were collected at an average depth of ~120.00‐fold, indicating high quality sequencing. Our evaluation identified a heterozygous nonsense mutation in *FAM83H* (NM_198488.5: c. 1819G > T) in the proband. This novel mutation introduces a premature stop codon at amino acid position 607 (p.(Glu607*)), leading to a C‐terminally truncated protein lacking 573 residues. Importantly, the conserved DUF1669 domain (located at the N‐terminus, residues 1–280) remains intact in the mutant protein, consistent with previous reports that FAM83H truncations associated with ADHCAI retain the N‐terminal domain (Tachie‐Menson et al. 2020; Wang et al. 2016). The mutation instead ablates the C‐terminal region, which is critical for proper cytoplasmic localization (Figure 3B). Segregation analysis by Sanger sequencing confirmed that the mutation followed an autosomal dominant mode of inheritance with complete penetrance. It was detected in the affected proband, her mother (Ⅱ:1), and grandmother (Ⅰ:1). In contrast, the wild‐type allele was present in the unaffected family members (Ⅱ:11 and III:2) (Figure 3A). The detected variant (NM_198488.5: c.1819G>T, p.(Glu607*)) has been submitted to ClinVar (accession no. SCV007613817) and classified as Pathogenic based on ACMG criteria, including evidence codes PVS1, PM2, PP1_Strong, and PS4_Moderate.

### 3.4 Bioinformatic Analysis

**FIGURE 4:**
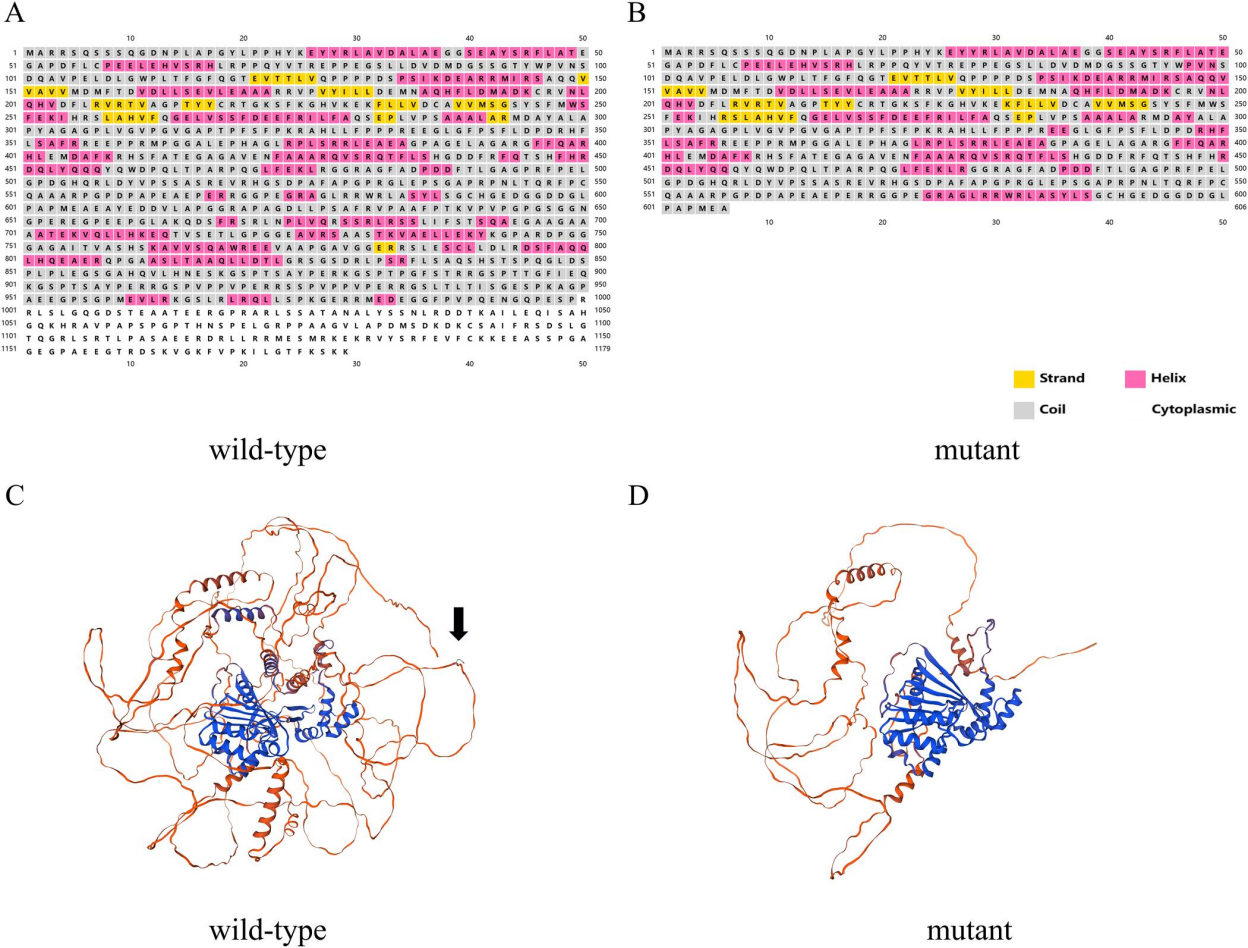
Predicted secondary and tertiary structures of the wild‐type and mutant FAM83H proteins. (A) Secondary structure of the wild‐type FAM83H protein. (B) Secondary structure of the mutant FAM83H protein, which is truncated and 573 amino acids shorter than the wild‐type. Helices, strands, coils, and cytoplasmic are presented pink, yellow, gray, and white boxes, respectively. Tertiary structures of the wild‐type (C) and mutant (D) FAM83H proteins. The mutation site is indicated by a black arrow.

Computational modeling provided strong evidence for functional disruption: MutationTaster predicted a disease‐causing probability of 0.99. The secondary and tertiary structures were predicted using the online programs PSIPRED Protein Sequence Analysis Workbench and SWISS‐MODEL, respectively. The results revealed structural transformations between the wild‐type protein (Figure 4A,C) and the truncated protein (Figure 4B,D), in addition to the 573 amino acids missing from the C‐terminus in the truncated protein. It should be noted that large regions of FAM83H, particularly the N‐terminal DUF1669 domain and surrounding sequences, are predicted to be intrinsically disordered (Bozatzi and Sapkota 2018). Therefore, the structural models presented here (Figure 4) should be interpreted as provisional and primarily illustrative of gross conformational changes rather than precise atomic details.

### 3.5 Subcellular Localization of the FAM83H Mutant

**FIGURE 5:**
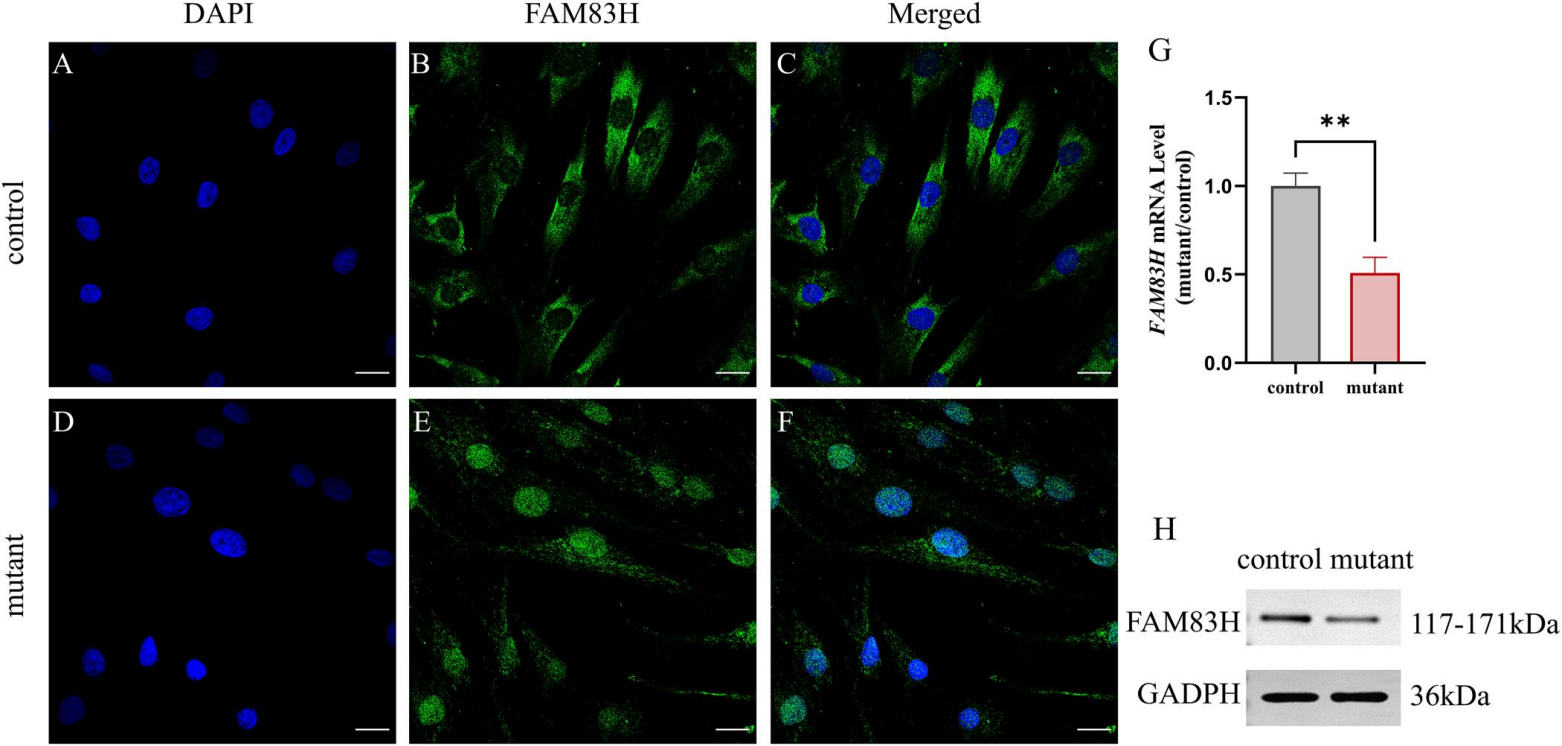
Subcellular localization of the FAM83H protein in both the control and mutant groups. (A–C) Confocal microscopy images showing uniform cytoplasmic distribution of the FAM83H protein (green) in the control group. Scale bar: 10 μm. (D–F) In the mutant group, the FAM83H protein (green) is predominantly localized in the nucleus. Scale bar: 10 μm. Nuclei are stained with DAPI‐containing Fluoroshield^TM^ mounting medium (blue). (G) RT‐qPCR analysis showed that the expression level of *FAM83H* mRNA was significantly lower in the mutant group (right) than in the control group (left). **p ˂ 0.01, *t*_(4)_ = 7.415. Consistent with this, western blot analysis confirmed a substantial reduction in FAM83H protein expression in the mutant group (H).

FAM83H normally localizes to the cytoplasm in a dispersed manner under physiological conditions (Lee et al. 2011). The p.(Glu607*) FAM83H mutation truncates 573 amino acids from the C‐terminus; this mutation may interfere with normal FAM83H protein function, including its subcellular distribution. We performed immunofluorescence to locate the FAM83H protein in PDLCs extracted from the proband. Unlike the uniform cytoplasmic distribution of the FAM83H in the control (Figure 5A–C), the Glu607* mutant showed predominant accumulation in the nucleus and minimal signal in the cytoplasm (Figure 5D–F).

### 3.6 Effects of the FAM83H Variant on Gene and Protein Expression

**FIGURE 6:**
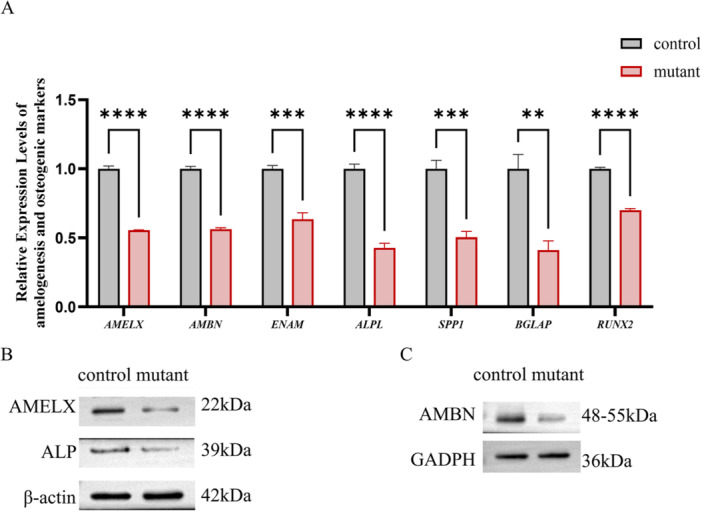
Expression levels of amelogenesis‐ and osteogenesis‐related genes and proteins in control and mutant groups. (A) RT‐qPCR analysis shows that the expression levels of enamel extracellular matrix genes (*AMELX*, *AMBN*, and *ENAM*) and osteogenic genes (*SPP1*, *BGLAP*, *ALPL*, and *RUNX2*) are significantly lower in mutant PDLCs compared to the control group, **p < 0.01, ***p < 0.001, ****p < 0.0001. (unpaired two‐tailed Student's *t*‐test, *df* = 4 for all comparisons) (B, C) Western blot analysis demonstrates reduced protein levels of AMELX, AMBN, and ALP in mutant cells.

RT–qPCR and western blot analysis revealed that the expression levels of *FAM83H* mRNA and protein were significantly lower in the PDLCs of ADHCAI patients than in controls (Figure 5G,H). Moreover, reduced FAM83H expression was associated with decreased expression of EMPs such as *AMELX*, *AMBN*, and *ENAM*, and the osteogenesis markers, including osteocalcin (*BGLAP*), osteopontin (*SPP1*), ALP **(** *ALPL*), and *RUNX2* (Figure 6A). Western blot analysis confirmed that the protein expression levels of AMELX, ENAM, and ALP were decreased in mutant cells (Figure 6B,C), supporting a dual regulatory role of FAM83H in matrix synthesis and mineralization.

## 4 Discussion

Our study identified a previously unreported *FAM83H* nonsense mutation (c.1819G>T: p.(Glu607*)) in a three‐generation Chinese pedigree with ADHCAI. This mutation introduces a premature stop codon that truncates the C‐terminal 573 residues. Functional analyses demonstrated that this mutation triggers pathogenic nuclear mislocalization of FAM83H, induces ultrastructural enamel defects, and suppresses the expression of key enamel matrix and osteogenic proteins, thereby providing a mechanistic explanation for the observed clinical phenotype.

The phenotypic spectrum of ADHCAI caused by *FAM83H* mutations exhibits considerable heterogeneity (Smith et al. 2017). While our proband presented with classic generalized manifestations—including exfoliated enamel, exposed dark dentin, loose proximal contacts, malocclusion, as well as plaque accumulation and gingivitis—other reported cases show milder phenotypes (Wright et al. 2009). This variability suggests potential influences from genetic modifiers or allelic heterogeneity. Notably, all pathogenic mutations reported to date (summarized in Table 1) are clustered within exon 5 (Wang et al. 2021), including ours, which encodes critical functional domains, underscoring the indispensable role of the C‐terminus in enamel mineralization.

**TABLE 1:** Summary of previously identified *FAM83H* mutations associated with ADHCAI.

Number	cDNA	Protein	Localization of FAM83H	Reference
1	c.973 C > T	p.(Arg325*)	Nucleus	Kim et al. (2008)
2	c.1192 C > T	p.(Gln398*)	Not mentioned	
3	c.1243 G > T	p.(Glu415*)	Not mentioned	Lee et al. (2008)
4	c.891 T > A	p.(Tyr297*)	Not mentioned	
5	c.1380 G > A	p.(Trp460*)	Nucleus	
6	c.2029 C > T	p.(Gln677*)	Not mentioned	
7	c.1330 C > T	p.(Gln444*)	Not mentioned	Hart et al. (2009)
8	c.1366 C > T	p.(Gln456*)	Not mentioned	
9	c.1354 C > T	p.(Gln452*)	Not mentioned	Hyun et al. (2009)
10	c.1408 C > T	p.(Gln470*)	Not mentioned	Wright et al. (2009)
11	c.860 C > A	p.(Ser287*)	Not mentioned	
12	c.1379 G > A	p.(Trp460*)	Not mentioned	
13	c.2080 G > T	p.(Glu694*)	Not mentioned	
14	c.923_924delTC	p.(Leu308Argfs*323)	Not mentioned	
15	c.1872_1873delCC	p.(Leu625Profs*703)	Not mentioned	
16	c.1374 C > A	p.(Tyr458*)	Not mentioned	El‐Sayed et al. (2010)
17	c.906 T > A	p.(Tyr302*)	Not mentioned	Haubek et al. (2011)
18	c.1993C>T	p.(Gln665*)	Not mentioned	Lee et al. (2011)
19	c.906 T > G	p.(Tyr302*)	Not mentioned	Song et al. (2012)
20	c.924dupT	p.(Val309Argfs*324)	Not mentioned	
21	c.1024 T > A	p.Ser342Thr	Not mentioned	Pourhashemi et al. (2014)
22	c.1669 G > T	p.Gly557Cys	Not mentioned	Urzúa et al. (2015)
23	c.1387 C > T	p.(Gln463*)	Not mentioned	Kantaputra et al. (2016)
24	c.1369 C > T	p.(Gln457*)	Not mentioned	Wang et al. (2016)
25	c.1915A>T	p.(Lys639*)	Not mentioned	
26	c.1282 C > T	p.(Gln428*)	Not mentioned	Prasad et al. (2016)
27	c.931dupC	p.(Val311Argfs*13)	Nucleus	Xin et al. (2017)
28	c.1130_1131delinsAA	p.(Ser377*)	Nucleus	
29	c.1147 G > T	p.(Glu383*)	Nucleus	
30	c.1261 G > T	p.(Glu421*)	Not mentioned	Nowwarote et al. (2018)
31	c.1222 A > T	p.(Lys408*)	Nucleus	Yu et al. (2018)
32	c.1309_1311delinsTAA	p.(His437*)	Not mentioned	Wang et al. (2021)
33	c.1375 C > T	p.(Gln459*)	Not mentioned	
34	c.1828G>T	p.(Glu610*)	Not mentioned	
35	c.1975G>T	p.(Glu659*)	Nucleus	Bai et al. (2022)
36	c.1289 C > A	p.(Ser430*)	Not mentioned	Alvarez et al. (2022)
37	c.1363 C > T	p.(Gln455*)	Not mentioned	Song et al. (2022)
38	c.2363 G > A	p.Ser788Asn	Not mentioned	Leban et al. (2022)
39	c.930_939dup	p.(Val314Argfs*14)	Not mentioned	Bloch‐Zupan et al. (2023)
40	c.1436 G > A	p.Gly479Asp	Not mentioned	Wredenhagen et al. (2023)
41	c.1055 C > A	p.(Ser352*)	Not mentioned	Kamps et al. (2025)
42	c.2137 C > T	p.(Gln713∗)	Not mentioned	Hany et al. (2025)

Western blot analysis confirmed the expression of a truncated FAM83H protein in mutant cells (Figure 5H), which aberrantly accumulated in the nucleus. This mislocalization represents a central driver of pathogenesis, likely through a dominant‐negative mechanism as described by Tachie‐Menson (Tachie‐Menson et al. 2020), whereby the mutant protein sequesters wild‐type FAM83H and/or its critical partners into non‐functional nuclear aggregates. The nuclear accumulation we observed provides a plausible explanation for the transcriptional downregulation of enamel matrix and osteogenic genes. Furthermore, this finding aligns with the model proposed by Yang (Yang et al. 2018), in which mutant FAM83H mislocalizes *CSNK1A1* to the nucleus, potentially disrupting its normal cytoplasmic functions. Although not directly tested here, such a mechanism could offer a unifying explanation for the aberrant signaling and gene expression patterns that ultimately lead to failed mineralization. This gain‐of‐function mechanism is corroborated by animal models; unlike *Fam83h* knockout mice, which exhibit normal enamel (Wang et al. 2016), mice expressing C‐terminal truncated *Fam83h* recapitulate the human disease (Wang et al. 2019), confirming that the phenotype is driven by the aberrant protein rather than haploinsufficiency.

These molecular perturbations culminate in the structural enamel deficiencies observed in our SEM‐EDX analysis. The decreased calcium content and significantly reduced Ca/P ratio (below the ideal value of 1.6; Nurbaeva et al. 2017) indicate incomplete mineralization. This stoichiometric imbalance compromises hydroxyapatite crystal stability, leading to a porous microstructure with disorganized enamel prisms. As a result, the enamel exhibits diminished hardness and a lower elastic modulus, accounting for its clinical susceptibility to attrition (Gjørup et al. 2009; Hyun et al. 2009; Wright et al. 1993). The increased porosity also facilitates pigment penetration—causing discoloration—and, following enamel loss, allows fluid flow within exposed dentinal tubules, underlying the symptoms of dentin hypersensitivity (Liu et al. 2020). Thus, the elemental deficiencies directly explain the structural and clinical hallmarks of ADHCAI.

Beyond elucidating structural defects, our study provides novel cellular insights by demonstrating that patient‐derived PDLCs carrying the *FAM83H* mutation exhibit coordinated downregulation of both enamel matrix (*AMELX*, *AMBN*, *ENAM*) and osteogenic markers (*RUNX2*, *BGLAP*, *SPP1*, *ALPL*). This dual suppression suggests that FAM83H acts as a master regulator of biomineralization. The concerted downregulation of key matrix proteins—responsible for hydroxyapatite nucleation (AMELX, Cho et al. 2014), elongation (ENAM, Hu et al. 2008), and ameloblast adhesion (AMBN, Fukumoto et al. 2004)—points to a critical failure during the presecretory phase of amelogenesis. Based on these, we propose a working model: truncated FAM83H may disrupt mineralization in part by suppressing matrix protein synthesis during the presecretory phase, thereby producing a compositionally flawed enamel matrix that is destined for mechanical failure after eruption. However, we caution that this model is derived primarily from patient‐derived PDLCs and requires validation in ameloblast‐like cells or *in vivo* models.

These findings should be interpreted in light of several limitations. Our functional analyses were derived from a single proband and relied primarily on patient‐derived PDLCs. While PDLCs offer a valuable and accessible *in vitro* system for mechanistic exploration, they are not enamel‐forming ameloblasts, and our findings should therefore be interpreted with appropriate caution. The relatively small sample size—one affected proband compared with three unaffected controls—further limits the generalizability of our conclusions. To fully validate the proposed transcriptional regulatory functions of FAM83H, future studies using ameloblast‐like cell lines (e.g., LS8 cells) or transgenic animal models expressing the p.(Glu607*) mutation will be essential. Despite these caveats, our findings offer new insights into the molecular pathology of ADHCAI and provide a foundation for further mechanistic and therapeutic exploration.

In conclusion, we have identified and characterized a novel ADHCAI‐associated *FAM83H* mutation. Through integrated genetic, cellular, and ultrastructural analyses, we delineate a coherent pathogenic model in which C‐terminal truncation precipitates a critical mislocalization of the mutant protein to the nucleus. This aberrant localization likely disrupts transcriptional regulation, culminating in the dual suppression of enamel matrix and osteogenic gene programs. The resulting deficiency in the organic matrix framework, combined with the elemental imbalances and ultrastructural defects revealed by SEM‐EDX, provides a compelling explanation for the formation of enamel that is intrinsically fragile and susceptible to the rapid post‐eruptive breakdown characteristic of ADHCAI. Therefore, our work expands the mutational spectrum of *FAM83H* and provides preliminary evidence for transcriptional dysregulation in patient‐derived cells, and contributes to the understanding of ADHCAI pathogenesis. Our findings highlight the potential importance of pre‐eruptive transcriptional events in ADHCAI, suggesting new directions for therapeutic exploration.

## Author Contributions

**Yanyu Huang** and **Yue Wang:** conceptualization. **Yue Wang** and **Hongfei Chen:** Formal analysis. **Yue Wang, Hongfei Chen,** and **Jiyong Lai:** investigation. **Yanyu Huang** and **Xueqing Huang:** methodology. **Yue Wang:** writing – original draft. **Yanyu Huang** and **Xueqing Huang:** writing – review and editing.

## Ethics Statement

This study was approved by the Ethics Committee of the School and Hospital of Stomatology, Sun Yat‐sen University (Approval No. KQYC‐2023‐39‐02). Written informed consent was obtained from all individual participants included in the study.

## Conflicts of Interest

The authors declare no conflicts of interest.

## Additional Declarations

We confirm that this work, in whole or in part, has not been under consideration for publication by any other primary scientific journal. All authors have read and approved the final manuscript.

## Supporting information


Supporting File 1

Supporting File 2

## Data Availability

The data that support the findings of this study are available on request from the corresponding author. The data are not publicly available due to privacy or ethical restrictions.
