# Supplementary material for: A Novel FAM83H Truncation Mutation Disrupts Enamel Mineralization in Autosomal Dominant Hypocalcified Amelogenesis Imperfecta
Source: Clin Exp Dent Res. 2026 Aug 13;12(4):e70431. doi: 10.1002/cre2.70431 (PMC13472511; doi:10.1002/cre2.70431)
Supplement: Supplementary file 1 [file CRE2-12-e70431-s001.docx]

Table S1 PCR primers for *FAM83H* gene

| Primers | Forward primer (5’-3’) | Reverse primer (5’-3’) |
| --- | --- | --- |
| E1-1 | CTCTGAGCTGGCCTGAGG | AGCAAGGACCAGCACCAG |
| E1-2 | CACAGGCAGCCTCGAGAC | ATCCCTAGGTTGCTGCAGTG |
| E3 | CCCCTAGTCATGTGTCTCCC | CACTACCCTCAAGCCCAAGA |
| E4 | GAGGGGAGGCTGGACAATG | GGCCCTGGTGTGGAAAGG |
| E5-1 | CTTGCAGACCTCACATCCCT | TTGAAGGCGTCCATATCCAG |
| E5-2 | CTTCCCCTCCTTCCTCGAC | GCCCAGGGTGAAGTCATC |
| E5-3 | CTACCAGCAGCAGTACCAGT | AGCCAGCACGTCGTCTTC |
| E5-4 | GTTTGGCCTCCTACTTGAGC | TGTACTTCTCCAGCAGCTCC |
| E5-5 | GGAGCTGTTGGAGAAGTACA | GCTCCCCTTCTGCTCGATAA |
| E5-6 | CCACCTCGGCTTACCCTG | GAATGGCCTTCGTGTCATCC |
| E5-7 | ATGGAGGATGAGGGTGGCTT | CTGCTGGCCTCCTCTTTCTT |
| E5-8 | GDTTCGAGGTCTTCTGCAAG | AATCCCAGCTACTCAGGAGG |
| E5-9 | ATTCCTCATTCTCTGGGCCC | TCCACAACCAGCCCTTCTAG |
| E5-10 | ACCACGTGTGTAGAAAGCCC | CACGTGGTTAAAGGCTGCAG |
