# Supplementary material for: A Novel FAM83H Truncation Mutation Disrupts Enamel Mineralization in Autosomal Dominant Hypocalcified Amelogenesis Imperfecta
Source: Clin Exp Dent Res. 2026 Aug 13;12(4):e70431. doi: 10.1002/cre2.70431 (PMC13472511; doi:10.1002/cre2.70431)
Supplement: Supplementary file 2 [file CRE2-12-e70431-s002.docx]

Table S2 Primers used for RT-qPCR

| Gene | Forward primer (5’-3’) | Reverse primer (5’-3’) |
| --- | --- | --- |
| *GADPH* | CAAAGTTGTCATGG ATGACC | CCATGGAGAAGGCTGGGG |
| *FAM83H* | AACCTGACCCAGCGCTTC | GAAGGCCGAGGGGAGCA |
| *AMELX* | GCAGCCTCATCACCACATCC | CAGGAACGGGCATCATTGG |
| *AMBN* | CTCTCCCATCACAGCCATCC | GCTTCTTTCAGTGCTGTGGC |
| *ENAM* | GGGGAGAACCAAGACACCAG | GGCCTGGTAGGATGCTTGTT |
| *SPP1* | TGATGCCACAGATGAGGACCT | CAGAGGGCATGCTCAGAAGC |
| *BGLAP* | GGCCCTGAGTCTGACAAAGC | CCCGGAGTCTGTTCACTACCTT |
| *ALPL* | TCCTGACCAAAAACCTCAAAGG | TGCTTCATSCAGAGCCTSC |
| *RUNX2* | GAACCAAGAAGGCACAGAC | AATGCGCCCTAAATCACTG |
